# Lab-to-Field Transition of RNA Spray Applications – How Far Are We?

**DOI:** 10.3389/fpls.2021.755203

**Published:** 2021-10-15

**Authors:** Aline Pereira Rank, Aline Koch

**Affiliations:** Institute of Phytomedicine, University of Hohenheim, Stuttgart, Germany

**Keywords:** RNAi-based plant protection, RNA biopesticides, dsRNA, siRNA, nanomaterial-based siRNA delivery, spray-induced gene silencing (SIGS)

## Abstract

The drastic loss of biodiversity has alarmed the public and raised sociopolitical demand for chemical pesticide-free plant production, which is now treated by governments worldwide as a top priority. Given this global challenge, RNAi-based technologies are rapidly evolving as a promising substitute to conventional chemical pesticides. Primarily, genetically modified (GM) crops expressing double-stranded (ds)RNA-mediating gene silencing of foreign transcripts have been developed. However, since the cultivation of GM RNAi crops is viewed negatively in numerous countries, GM-free exogenous RNA spray applications attract tremendous scientific and political interest. The sudden rise in demand for pesticide alternatives has boosted research on sprayable RNA biopesticides, generating significant technological developments and advancing the potential for field applications in the near future. Here we review the latest advances that could pave the way for a quick lab-to-field transition for RNA sprays, which, as safe, selective, broadly applicable, and cost-effective biopesticides, represent an innovation in sustainable crop production. Given these latest advances, we further discuss technological limitations, knowledge gaps in the research, safety concerns and regulatory requirements that need to be considered and addressed before RNA sprays can become a reliable and realistic agricultural approach.

## Introduction

Modern agriculture faces diverse challenges related to the fact that the world population is expected to rise by 26% by 2050. Given this projection, agriculturists must boost productivity to meet emerging food needs (United Nations Department of Economics and Social Affairs, 2019). Major challenges to these production levels arise due to agricultural pests. Pests contribute estimated losses of up to 40% of crops worldwide, with insects serving as causal agents of damage and disease transmission (Douglas, 2018). So far, plant protection primarily relies on the use of chemical pesticides. However, the use of conventional pesticides contributes to soil, water, and air pollution and is predicted to be a major driver of an alarming loss of biodiversity worldwide (Wagner et al., 2021). In addition, improper and controlled chemical treatments detrimentally affect the environment, consumers, and users. Moreover, perennial applications result in the development of pesticide resistance in pathogens and pests (Fotoukkiaii et al., 2021).

Intergovernmental initiatives such as the EU’s farm-to-fork strategy (part of the European green deal) aim to rethink and redesign food systems, for example by developing frameworks for sustainable food production. A central goal is to reduce the overall use and risk of chemical pesticides by 50% by 2030 (European Commission, 2021). Given the social-political demand for an ideally pesticide-free agriculture, safe “green” alternatives are urgently desired to facilitate sustainability in crop protection. Thus, RNAi-based technologies are discussed as low risk pesticides that may enable reaching pesticide reduction and sustainability goals (Taning et al., 2021).

A promising alternative to chemical pesticides relies on harnessing the mechanistic of RNA interference (RNAi). Briefly, RNAi is a conserved cellular mechanism that regulates and protects eukaryotic cells against harmful nucleic acids. In this natural phenomenon, double-stranded RNAs (dsRNAs) are processed by Dicer enzymes into 21–24 nucleotide (nt) small interfering RNAs (siRNAs). These siRNAs either mediate transcriptional gene silencing (TGS) by ensuring inhibitory DNA and histone modifications as well as chromatin remodeling, or they facilitate post-transcriptional gene silencing (PTGS) by preventing translation of targeted mRNA transcripts. Both TGS and PTGS served as blueprints for the development and continuous improvement of RNAi toolkits. In agriculture, RNAi has proven to be an effective strategy in controlling pathogens such as viruses, bacteria, and fungi, as well as insect pests, mites, and nematodes (Fire et al., 1998; Baum et al., 2007; Zotti et al., 2018; Mezzetti et al., 2020; Koch and Wassenegger, 2021).

A decade ago, studies focused on the transgene-based endogenous expression and formation of dsRNA in host plants, termed host-induced gene silencing (HIGS) (Nowara et al., 2010). Nowadays, transgenic approaches are clouded by some major drawbacks. First, these approaches are laborious, complicated, and time-consuming, and their applicability is restricted by the transformability of the host plant. They are also very expensive; for example, the commercialization of a transgenic crop is estimated to reach 140 million dollars (Rosa et al., 2018). Finally, they are weakly accepted, as public distrust of GM plants persists, with an ongoing upward trend (Herman et al., 2021). Additionally, orders and species present completely different and variable demands, raising concerns about whether this technology is effective under field conditions (Scott et al., 2013; Yu et al., 2016; Cooper et al., 2019). Given this assessment, there is not only a need for alternatives that avoid the use of chemical pesticides but also increasing interest in finding alternatives to GM-based measures (Dalakouras et al., 2020; Das and Sherif, 2020). A breakthrough was achieved by demonstrating that dsRNAs effectively induce PTGS and confer disease resistance upon foliar spray (Nature, 2016), termed spray-induced gene silencing (SIGS) (Table 1; Koch et al., 2016; Wang et al., 2016). Compared to HIGS, SIGS is much faster, cheaper and easier to handle, and it might reach a broader range of hosts. Interestingly, a direct comparison of HIGS and SIGS revealed SIGS to be more efficient under lab conditions (Koch et al., 2019; Höfle et al., 2020). However, the lab-to-field transition will require optimization and further development of SIGS technology to increase stability (UV, rain) and specificity (off-target risks) under environmental conditions. In this review, we will highlight the most promising attempts to further develop SIGS for its lab-to-field transition and discuss significant achievements in improving stability and reducing environmental risks. In addition, we provide an overview of RNA spray approaches and their particularities, including nanomaterial-based delivery, efficiencies and durability.

**TABLE 1:** Summary of RNA spray studies in plants.

	**Target organism**	**Target gene (dsRNA length)**	**Plant species**	**Nanoformulation**	**Applied dsRNA (μ g)**	**Application method**	**Efficacies**	**Durability**	**References**
**Viruses**	Sugarcane Mosaic Virus (SCMV)	Coat protein (∼150 bp)	*Zea mays*	*Escherichia coli* HT115 (DE3)	3 μg/L	Spray of bacteria-produced dsRNA	Total inhibition of virus infection	30 days post inoculation (dpi)	Gan et al., 2010
	Pepper mild mottle virus (PMMoV) Cucumber mosaic virus (CMV)	PMMoV Replicase (977 bp) CMV Replicase (2b) (330 bp)	*Vigna unguiculata Nicotiana tabacum*	Layered double hydroxide (LDH) clay nanosheets	1.25 μg of dsRNA and/or 3.75 μg of LDH	Sprayed with an atomizer	Total inhibition of virus infection (systemic protection)	Up to 20 days after a single spray	Mitter et al., 2017
	Bean common mosaic virus (BCMV)	Nuclear inclusion b protein (480 bp) Coat protein (461 bp)	*Nicotiana benthamiana Vigna unguiculata*		100 μg	Mechanical inoculation with carborundum as an abrasive and sprayed with an atomizer	Reduction in infection to 45% and to 8.3%	Analyzed for up to 10 days	Worrall et al., 2019
	Tomato yellow leaf curl virus (TYLCV)	Coat protein (CP) (680–700 bp)	*Nicotiana benthamiana Solanum lycopersicum*		1.25 μg of pDNA, 3.75 μg of LDH	Sprayed using an atomizer at ∼125 μl/cm^2^	Delivered systemically. Lower incidence and severity. Symptom expression rate reduced to 41.7%	Up to 35 days	Liu et al., 2020
**Insects**	*Sitobeon avenae*	Salivary sheath protein (491bp)	*Hordeum vulgare*	Naked dsRNA	20 ng/μl	Foliar spray on leaves	Reduced transcript level of 60%	Monitored up to 5 days	Biedenkopf et al., 2020
	*Leptinotarsa decemlineata*	Inhibitor of apoptosis, Actin; HSP70; Dynamin (300–600 bp)	*Solanum tuberosum*	*Escherichia coli* HT115 (DE3)	15 μl of 0.85 μg/μl (larvae) and 2.5 μg/μl (adults) dsRNA spread on each disk surface. Expressed by bacteria heat−killed in 4ml sprayed	dsRNA spread on each disk surface and dried for 10 min	∼100% larval mortality (dsIAP or dsActin); 80% adult mortality (dsActin) Feeding CPB with heat−killed bacteria induced significantly higher mortality (70–90%)	Mortality recorded until 11th day and up to 6th for the bacteria expressed assay	Máximo et al., 2020
		Actin (297bp)	*Solanum tuberosum*	naked dsRNA	12 μL (100, 30, 10, 3, 1, and 0.3 ng)	Purpose-built spraying device on potato leaf disks (Ø = 2 cm)	German strain and Spanish field strain E02 almost 100% mortality (30 ng dsActin). Spanish strain E01 showed only 30% mortality. By day four > 95% of the exposed larvae were dead	Monitored up 5 days	Mehlhorn et al., 2020
		*Mesh* gene (417 bp)	*Solanum tuberosum*	Naked dsRNA	10 μg/ml.	25 m^2^ plots of potato plants were sprayed under field conditions	Observed field mortality was slightly lower compared to laboratory trials	Monitored up 13 day	Petek et al., 2020
	*Phaedon cochleariae*	Cactus, srp54k, rop, α-SNAP Shibire, PP-α, hsc70–3, rpn7, rpt3; (317 and 599 bp)	*Brassica oleracea*	Naked dsRNA	0.3 μg (9.6 g/ha), 1 μg (32 g/ha) to 3 μg (96 g/ha)	Custom-built spraying device, multi-well plate foliar RNAi screening procedure	Suppression of transcript level rpt3 (94.5%), srp54k (94.1%), rpn7 (93.9%), α-SNAP (84.9%), shibire (81.3%), PP-α (80.5%) hsc70–3 (75.9%)	Monitored over 10 days	Mehlhorn et al., 2021
	*Helicoverpa armigera*	Juvenile hormone methyltransferase, Acetylcholine esterase; (21 nt)	Chickpea	Chitosan nanoparticles (CNPs)	One milliliter of CNPs-dsRNA (200 μg:1,000 ng wt/wt)	Sprayed over the plant canopy with a hand-held mist sprayer	100% insect mortality	Monitored up to 5 days	Kolge et al., 2021
	*Henosepilachna vigintioctopunctata*	Ecdysone receptor (EcR)	*Solanum tuberosum*	*Escherichia coli* HT115 (DE3)	0.5 μg/mL	dsEcR-immersed foliage and dsEcR-*E. coli* directly sprayed to the foliage of greenhouse-growing potato plants	Only 40% of the treated larvae formed Pupae (6–8 days). Of them, 60% exhibited a defective phenotype	Monitored for up to 10 days	Wu et al., 2021
**Fungi**	*Fusarium graminearum*	Cytochrome P450 lanosterol C-14α-demethylases (CYP51A, CYP51B, CYP51C) (CYP3RNA) (791 nt; 21–24 nt)	*Hordeum vulgare*	Naked dsRNA	dsRNA was diluted in 500 μL water to a final concentration of 20 ng/μL	Detached barley leaves were sprayed using a spray flask	Reduction of transcript level: 72% (CYP51A), 90% (CYP51B), and 71% (CYP51C); inhibition of fungal growth	Analyzed for up to 8 days	Koch et al., 2016
	*Botrytis cinerea Verticillium* spp.	Dicer-like (DCL)1 DCL2 (315 bp)	*Arabidopsis thaliana Nicotiana tabacum Solanum lycopersicum Fragaria rosales Vitis vinifera Lactuta sativa Allium cepa Rosaceae*	naked dsRNA	20 ng/μL	dsRNA were dropped onto the surface of fruits, vegetables and rose petals and *B. cinerea* Inoculum was applied on the same spot	Reduced fungal growth	Analyzed for up to 8–10 days after	Wang et al., 2016
	*Fusarium asiaticum*	Myosin5 (Myo5) (∼500 bp)	*Triticum aestivum*	Naked dsRNA	400 ng of fluorescein-Myo5-8 dsRNA	Sprayed using a spray flask	Inhibition of mycelial growth (31–70%), interference in life cycle and virulence, cell wall defects, life cycle disruption and virulence reduction	9 h unless the dsRNA was continuously supplied	Song et al., 2018
	*Fusarium graminearum*	294 nt (FgCYP51A) 220 nt (FgCYP51B) 238 nt (FgCYP51C) 514 nt (FgCYP51A/CYP51B) 458 nt (FgCYP51B/CYP51C) 532 nt (FgCYP51A/CYP51C)	*Hordeum vulgare*	Naked dsRNA	dsRNA was diluted in 500 μL water to a final concentration of 20 ng/μL	Barley leaves were detached and sprayed using a spray flask	Reduced fungal growth and transcript level to less than 10%	Monitored up to 5 days	Koch et al., 2019
		DICER-like1 and 2; ARGONAUTE1 and 2; AGO-interacting protein FgQIP; RecQ Helicase;RNA-dependent RNA polymerases (∼1,000 bp)					Central role in different steps of sexual and asexual reproduction, in fungal pathogenicity and DON production		Gaffar and Koch, 2019
		500–, 800–, 1,518 nt (FgCYP51A) 400–, 800–, 1,575 nt (FgCYP51B) 400–, 800–, 1,548 nt (FgCYP51C)					Inhibition of fungal infection symptoms up to 82%		Höfle et al., 2020
		365 nt, 1,529 nt (FgAGO1/AGO2) 355 nt, 1,570 nt (FgAGO1/DCL1) 366 nt, 1,528 nt (FgAGO1/DCL2) 374 nt, 1,783 nt (FgAGO2/DCL1) 1,741 nt (FgAGO2/DCL2) 1,782 nt (FgDCL1/DCL2)					Inhibition of fungal infection up to 60%; reduced transcript level up to 79%		Werner et al., 2020
	*Phakopsora pachyrhizi*	Acetyl-CoA acyltransferase 40S ribosomal protein S16, Glycine cleavage system H protein; (200–400 bp)	*Glycine max*	Diethyl-pyrocarbonate	1 ml of diluted dsRNA (20 μg dsRNA)	Each box (six detached individual leaflets) was evenly sprayed	Average of over 73% reduction of pustule numbers 75% reduction in biomass accumulation	Monitored up to 2 weeks	Hu et al., 2020
	*Phytophthora infestans*	Sorbitol dehydrogenase, Translation elongation factor 1-α, Phospholipase-D like 3, Glycosylphosphatidylinositol-anchored acidic serine-threonine rich HAM34-like protein, Heat shock protein-90; (402bp-536bp)	*Solanum tuberosum*	*Escherichia coli* HT115 (DE3) 0.5% Nanoclay solution (5, 10, and 20 ppm)	100–, 150–, 250– and 500 ng and 1 μg dsRNA	Sprayed with dsRNA-nano clay formulation using automizer	Reduction in growth, sporulation and symptom expression, 15 days, control collapsed and wilted while dsRNA nano clay sprayed plants were erect and healthy	15 days	Sundaresha et al., 2021
	*Botryotinia fuckeliana*	Chitin synthase class III and DCL1 and DCL2	*Fragaria ananassa*	*Escherichia coli-*derived anucleated minicells	125–1,000 ng/ml	Topical spray application	Selectively knocked-down the target genes and led to significant fungal growth inhibition *in vitro*. Compensatory relationship between DCL1 and DCL2 gene transcripts	12 days	Islam et al., 2021
	*Fusarium oxysporum*	CYP51, chitin synthase 1, Elongation factor 2 (732bp)	*Solanum lycopersicum*	Layered double hydroxide nanosheets	300 μg of dsRNAs in 3 mL of _*dd*_H_2_O per plant	Spraying on plant leaves avoiding stems	Reduced fungal growth dsCYP51showed 93% reduction in transcript abundance	Monitored up to 8 days	Mohamed and Youssef, 2021
	*Phytophthora infestans*	Guanine-nucleotide binding protein β-subunit, haustorial membrane protein, cutinase, endo-1,3(4)-β-glucanase (436 bp)	*Solanum tuberosum*	Naked dsRNA	20 ng/μL	dsRNA sprayed on potato leaves in a detached leaf assay	Decreased disease progression, smaller and aberrant Mycelial phenotype	5 days	Kalyandurg et al., 2021
**Plants**	*Nicotiana benthamiana*	Green fluorescence protein (GFP), (21–, 22–, and 24 nt)	*Nicotiana benthamiana*	Naked dsRNA		High-pressure spraying procedure (HPSP)	Local and systemic silencing exception of siR24, delayed and weak local silencing (10 dpa), other GFP siRNAs induced local silencing 2 dpa	20 days post application	Dalakouras et al., 2016
		GFP (322, 139 bp)			200 μl of dsRNA midGFP (10, 20, 200, and 240 ng/μl) 200 μl of dsRNA-5’GFP (24, 48, and 240 ng/μl)		None of the samples sprayed with dsRNA-midGFP (0/15), dsRNA-5’GFP (0/9) or water (0/9) showed silencing up to 3 weeks after spraying	–	Uslu et al., 2020
		(CaMV) 35S promotor (333bp)		Naked dsRNA	50 μg in 500 μl	High-pressure spraying (carborundum was added)	Methylation of the 35S promotor was observed 10 days after spraying	10 days post spraying	Dalakouras and Ganopoulos, 2021
		GFP (124 bp)		Carbon dots (CD) surfactant BreakThru S279 was added to the CD-dsRNA complexes at a final concentration of 0.4% (v/v)	12 ng/μL (3.8 μL/cm^2^, 45 ng/cm^2^)	Spray application was done with Iwata HP-M1 handheld airbrush sprayer with air pressure set to 82 kPA (∼12 PSI)	MgCheH transcript levels showed a 79% reduction in the phenotypic tissues at five days after treatment. Reduction of 88% in GFP protein levels was observed	Monitored up to 12 days	Schwartz et al., 2020
		GFP (22 nt)			60 μl of solution/plant (3-4-leaf transgenic seedling)		No difference was observed in the extent or frequency of systemic silencing comparing the events containing or not the partial transposase. Expression was reduced to 48 and 72%.	Up to 14 days	Hendrix et al., 2021
		GFP (siRNA loading)		DNA nanostructures	100 nM for both the nanostructure and the siRNA duplex	Infiltrating nanostructures with a 1-ml needleless syringe and without using any surfactant.	40–59% (varies with DNA structures) in both mRNA and protein level	Internalization into plant cells 12h post-infiltration; gene silencing disappears 7days post-infiltration	Zhang et al., 2020
				Gold nanoclusters (AuNCs)	25 ng siRNA per 1 μg AuNCs		32–35% reduction in GFP 3 days postinfiltration	Internalization into plant cells 0,5-1 h post-infiltration	Zhang et al., 2021
				Single-walled carbon nanotubes	100 nM siRNAs		95–92% (mRNA level) ∼40% (protein level)	Internalization into plant cells 4 h post-infiltration	Demirer et al., 2020

## Major Challenges and Recent Achievements in Transferring RNA Spray to Field Environments

The immense potential of sprayable RNA biopesticides for meeting current agro-economic challenges has prompted the development of RNAi technology. Initial euphoria was a major driver leading to a rapid increase of reports, resulting in 54 studies over the last 3 years (PubMed, August 2021) demonstrating academic proof-of-concept for several pathosystems (Table 1). However, at the same time, it became obvious that the molecular mechanism for uptake, processing and transport of sprayed RNA biopesticides was inadequately understood.

Initially, only the GM-based HIGS approach was considered because transgene-derived dsRNA was thought to be robust and reliable in the delivery of PTGS inducers. Naked dsRNA showed effectiveness under lab conditions but had the potential for instability when sprayed in fields, where it could be degraded by UV radiation or washed off by rain. To circumvent this, several studies, inspired by human RNAi therapy research (Swaminathan et al., 2021), attempted to increase stability and durability by establishing protective envelops around dsRNAs and siRNAs (Pugsley et al., 2021; Yan et al., 2021). In mammals, the delivery of siRNA therapeutics is mediated by liposome encapsulation (Liu and Huang, 2021; Zabel et al., 2021). Nanoparticle- and other carrier-based delivery of dsRNA/siRNA has been widely used, with remarkable success, in start-up companies such as “RNAissance^1^” which focus on broad, cost-efficient topical RNAi uses in agriculture. Their final dsRNA products are advertised as safe, ready to use and stabler than naked dsRNAs. This advancement represents a step toward lowering the amount of applied RNA biopesticides since it is estimated that 2–10 g of dsRNA are required to protect 1 ha (Das and Sherif, 2020). However, the application amount and frequency depend on several largely unknown factors that determine dsRNA persistence, distribution and dilutional and degradational processes in host plants as well as target species. Moreover, it is indisputable that mechanistic insights, which determine strength and limitations in a pathosystem-specific manner, will be required to optimize and further develop RNA sprays, as well as to anticipate obstacles that will appear when transferring RNA sprays to field environments.

## Significant Advances of RNA Spray Controlling: Viruses

Given that the RNAi originally evolved as a primary antiviral defense in plants, it is not surprising that most studies have shown that RNAi is most effective at controlling viral pathogens. For example, across 75 studies, HIGS-based RNAi showed an average viral resistance of 90% (Gaffar and Koch, 2019; Koch and Wassenegger, 2021). Notably, similar efficacies were demonstrated when dsRNAs were applied exogenously (Table 1). Conclusive RNAi-based effects were reported in 2016 by Konakalla et al., who demonstrated that the exogenous application of dsRNA targeting the viral silencing suppressor *p126* gene and the *CP* gene of TMV conferred virus resistance in tobacco (Table 1). The authors further showed systemic spreading of dsRNA from local to systemic tissues within an hour of dsRNA application using semi-quantitative RT-PCR (Konakalla et al., 2016). They found that p126 dsRNA levels continuously decreased in the local treated tissue, from 3 to 9 dpi, until dsRNA was no longer detectable. Another study supported these findings, showing that exogenously applied dsRNA derived from the *HC-Pro* and *CP* genes of ZYMV protects watermelon and cucumber against ZYMV and that it spread systemically over long distances in cucurbits (Kaldis et al., 2018). Further emphasizing the systemic spread of RNA biopesticides, the movement of sprayed dsRNA from barley leaves to stems and root tissues was demonstrated within 3 days after spray treatment (Biedenkopf et al., 2020). Systemic distribution is of key importance because it indicates that RNA biopesticides could be promising substitutes for systemic pesticides. Moreover, translocation from leaves (application sites) to roots suggests that foliar sprays may be able to target soil-borne infections, thus circumventing the need to develop soil-specific RNA treatments.

Interestingly, the induction of virus resistance by exogenous application had already been achieved 20 years ago (Tenllado and Díaz-Ruíz, 2001). However, “exogenous” does not necessarily imply a spray application. Thus, we must distinguish between different application strategies, as most of them work on a lab scale but are unsuitable and unpracticable for field applications (Table 1).

As already indicated, numerous exogenous application strategies rely on preparatory treatments, such as the mechanical inoculation of leaves, to guarantee efficient dsRNA uptake (Table 1). The rubbing or dusting of leaf surfaces with Carborundum (silicon carbide) as an abrasive is widely used as a pretreatment (Tenllado and Díaz-Ruíz, 2001; Tenllado et al., 2003; Yin et al., 2009; Konakalla et al., 2016; Necira et al., 2021). Given the unsuitability of such pretreatments on the field scale, subsequent research, inspired by human RNAi therapeutics where nanotechnology is unavoidable, has attempted to formulate dsRNA for efficient and targeted RNA delivery. Such formulations can help to increase cellular uptake, improve stability (and thus overcome environmental degradation by UV radiation or surface wash-off) and provide long-term protection against the targeted pathogen. Toward this, the first breakthrough was achieved by Mitter et al. (2017) by using positively charged layered double hydroxide (LDH) clay nanosheets as a dsRNA carrier (BioClay). This technology was originally developed for the delivery of siRNA therapeutics to mammalian cells (Ladewig et al., 2009, 2010). The authors found that loading dsRNA on LDH prolonged durability on the leaf surface for 30 days and increased stability through protection from nuclease degradation (Mitter et al., 2017). Moreover, they showed the uptake of dsRNA into plant cells and induction of endogenous RNA silencing, which mediated systemic protection against the targeted VSR *2b* gene of CMV on cowpea and tobacco. In field BioClay allows sustained release of dsRNA on the leaf surface under ambient conditions (Ram Reddy et al., 2006; Xu et al., 2006; Reddy et al., 2008; Mitter et al., 2017). Notably, this study proved that the LDH nanocarrier can be completely degraded over time, thus resulting in a slow and sustained release of dsRNA under environmental conditions (Mitter et al., 2017). After this pioneering work on the biodegradability of clay-based nanomaterials, dsRNA-BioClay has been used to target the bean common mosaic virus (BCMV) to protect *N. benthamiana* and *Vigna unguiculata* (Worrall et al., 2019).

While this study provides a significant step forward in making RNA spray an applicable and sustainable approach for pathogen and pest control in agriculture, questions remain about how to produce efficient amounts of dsRNA for spray applications in field trials. Initially, *Escherichia coli*-based dsRNA production was used (Tenllado and Díaz-Ruíz, 2001; Tenllado et al., 2003; Yin et al., 2009; Gan et al., 2010) due to financial constraints and to the poor availability of suitable dsRNA synthesis kits. Interestingly, the latest studies showed that *E. coli* cannot only be used as a dsRNA factory but also provides adequate properties for dsRNA encapsulation (Islam et al., 2021; Necira et al., 2021). The authors of these studies concluded that the effects of *E. coli*-encapsulated dsRNA did not differ from the topical application of naked dsRNAs (Necira et al., 2021; Table 1). *E. coli*-encapsulation may nevertheless prove superior to naked dsRNA, as it provides a protective envelope conferring higher stability under field conditions, though host species are limited.

Numerous studies have addressed the question of how to reduce the production costs of dsRNA. Some have synthesized dsRNA using *in vitro* transcription kits, suitable for lab experiments but inconceivable for large-scale applications given their bad price-performance ratio ($700/mg). Thus, subsequent attempts have focused on large-scale production and on the purification of sprayable RNAs to make them commercially competitive and economically achievable, with great success. For example, recent research efforts have rapidly produced cost-effective, large-scale microbial-based dsRNA production using bacteria, such as *E. coli* (Voloudakis et al., 2015; Ahn et al., 2019; Bento et al., 2020; Niño-Sánchez et al., 2021) and *Pseudomonas syringae* (Niehl et al., 2018) and the yeast *Yarrowia lipolytica* (Timmons et al., 2001; Palli, 2014). Given these significant achievements, dsRNA costs per gram have dropped from $12,500 in 2008 to $100 in 2016, $60 in 2020 and finally $2 in 2021 (de Andrade and Hunter, 2016; Zotti et al., 2018; Dalakouras et al., 2020). Recently, large-scale cell-free production has further lowered the price to less than $0.50 per gram, making RNAi competitive in the market.^2^

Based on the plethora of proof-of-concept studies and recent achievements in nanomaterial-based dsRNA and siRNA delivery, which allow for highly effective virus control even under field conditions, fundamental knowledge on the molecular mechanisms and factors that determine uptake (over cuticle and cellular barriers), processing and translocation has begun to emerge.

## Significant Advances of RNA Spray Controlling: Fungi

Since 2010 the number of HIGS-based studies demonstrating fungal disease control has continued to increase (Nowara et al., 2010; Koch and Wassenegger, 2021). The first reports that showed that RNA spray can fight fungus were directed against two necrotrophic ascomycetes *Fusarium graminearum* (Koch et al., 2016) and *Botrytis cinerea* (Wang et al., 2016). These case studies further energized the debate on whether RNA spray is a realistic approach for future field applications. Beyond cost concerns, the obvious question was how to enhance uptake of RNA biopesticides by plants, thus avoiding degradation under environmental conditions. The uptake of dsRNA depends on leaf surface stability, and efficient and subsequent uptake can prevent premature degradation or wash-off by rain. Notably, sprayed RNAs must overcome several physical and cellular barriers to reach their cognate mRNA targets. First, they must overcome the “outside-inside” or cuticle barrier at the leaf surface, which is especially relevant when targeting pathogens that replicate and grow intra- or intercellularly, such as viruses and fungi, or sap-sucking insects like aphids or whiteflies. Like viral pathogens, fungi maintain an intimate relationship with their hosts, acquiring nutrients in a biotrophic, hemibiotrophic, or necrotrophic manner. Given their different lifestyles, fungi may take up sprayed RNAs from extra-, inter- as well as intracellular space. Passing through this barrier was assumed to be passively facilitated by stomata opening (Koch et al., 2016). Given this assumption, one could ask whether stomata density and leaf architecture represent limiting factors in RNA uptake. This would explain why SIGS works well for some plant species and not for others. Considering this, formulations that facilitate the opening of stomata or increase leaf permeability may help to increase dsRNA uptake from foliar sprays.

However, before sprayed dsRNA can enter plant cells for processing and translocation, they reach another barrier, the “apoplast-symplast” interface. Entering plant cells is necessary for processing into siRNAs by plant DCLs. However, in particular cases, the uptake of unprocessed dsRNA may also occur, depending on the lifestyle of the targeted pathogen. For example, SIGS of the necrotroph *F. graminearum* require the uptake of unprocessed dsRNA precursors and their subsequent processing by the fungal RNAi machinery (Koch et al., 2016; Gaffar et al., 2019). Based on these findings, it has been hypothesized that the uptake of long, unprocessed dsRNA and its processing into many different inhibitory siRNAs by the target organism might lead to higher gene silencing efficiencies and increased disease resistance (Koch et al., 2019). However, there is some controversy about the uptake of dsRNA versus siRNA, for instance in terms of the relative efficacies and off-target risks (Koch and Wassenegger, 2021). Interestingly, preliminary data suggest that if sprayed dsRNA is too long, the length might interfere with sufficient cellular uptake (Höfle et al., 2019). To prove whether uptake of unprocessed dsRNA really confers stronger effects, it will be helpful to compare SIGS efficacies of biotrophic and necrotrophic pathogens. So far, the disease resistance level achieved by SIGS has been comparable (Hu et al., 2020) or even superior (Höfle et al., 2019; Koch et al., 2019) to HIGS-based efficiencies under lab conditions.

Interestingly, a recent study that assessed the dsRNA uptake ability of different fungi revealed that dsRNA uptake efficiencies varied across the tested fungal and oomycete species (Qiao et al., 2021). For example, the authors demonstrated efficient dsRNA uptake for *Botrytis cinerea*, *Sclerotinia sclerotiorum*, *Rhizoctonia solani*, *Aspergillus niger*, and *Verticillium dahliae*. They found no uptake for *Colleotrichium gloeosporioides*, modest uptake for the non-pathogenic fungus *Trichoderma virens* and limited uptake for the oomycete *Phytophthora infestans* (Qiao et al., 2021). This study is of great significance, as it confirms and extends previous findings (Koch et al., 2016; Wang et al., 2016; McLoughlin et al., 2018) and further supports the idea that necrotrophic fungi exhibit a stronger response to exogenous RNA applications. Whether this is because they can take up unprocessed dsRNA, which may lead to higher gene silencing efficiencies - based on the finding that *F. graminearum* DCLs are required for SIGS (Koch et al., 2016; Gaffar et al., 2019) – or whether the overall amount of dsRNA absorbed in less time correlates with their necrotrophic lifestyle needs further verification. Supporting this idea, Hailing Jin and her team observed dsRNA uptake as early as 6 h after YFP-dsRNA *in vitro* treatment of the necrotrophs *B. cinerea*, *S. sclerotinia*, and *R. solani*, suggesting faster dsRNA uptake compared to *A. niger* (10 hpt) and *V. dahliae* (12 hpt). In addition, they demonstrated the antifungal activity of topically applied dsRNAs targeting fungal vesicle pathway genes or *DCL* genes in *B. cinerea*, *S. sclerotinia*, *R. solani*, and *A. niger* (Qiao et al., 2021). However, as the dsRNAs were dropped onto the surface of each plant or fruit sample and the fungi were applied directly to the dsRNA-treated area, we do not know from this study whether the uptake of plant DCL-processed siRNA would have made a difference. In other words, the type of RNA offered for uptake was limited to dsRNA, bringing us back to the initial question of whether the uptake of unprocessed dsRNA is a crucial determinant of SIGS efficacy, and thus whether the uptake of dsRNA by the plant represents a limiting factor regarding the durability of protective RNA spray effects.

Regarding this question, we need to differentiate between sprayed RNAs that stick to the plant (or fruit or vegetable) surface and those that enter plant cells. We predict that surface interaction of dsRNA or siRNA with microbial targets will require intense stabilization formulas, with potential risks for environmental accumulation and pollution. Cellular uptake, on the other hand, may have a protective effect, but promoting fast processing or even bearing the risk of enzymatic and lytic degradation together with dilution over distance. Although we seek RNA-stabilizing formulas, at the same time we question RNA degradability as a selling point. Recently, dsRNA spray effects against *B. cinerea* infection were found to last for 7 days after spraying onto tomato plants in a greenhouse, indicating a quite rapid degradation. Notably, the authors of this study concluded that dsRNA gets degraded by the environment, suggesting no cellular uptake by the plant (Qiao et al., 2021). Although short longevity can be determined by degradation, it remains unknown whether this degradation occurs outside or inside the sprayed plant.

So far, most SIGS studies demonstrating the control of fungal pathogens were conducted with naked, un-formulated dsRNA (Table 1). However, formulations that increase cellular uptake (especially for plants that showed no absorption of naked dsRNA from their surfaces) or surface stability (especially for post-harvest products such as fruits and vegetables) will boost broad applicability and the lab-to-field transition. A recent study showed that *E. coli*-derived anucleated minicells can be used as a cost-effective platform for dsRNA production and encapsulation, shielding dsRNA from RNase degradation (Islam et al., 2021). The authors demonstrated that the protection of strawberries from *Botryotinia fuckeliana* infection was prolonged to 12 days under greenhouse conditions, further emphasizing the added value of encapsulation for dsRNA stability. Although extending protection against the gray mold of tomatoes by 7 days (naked dsRNA) (Qiao et al., 2021) and strawberries by 12 days (Minicell-based) (Islam et al., 2021) already represent positive results, there is still room for further improvement. Thus, data on durability and persistence together with systemic distribution are highly desired for “lab-to-field” technology transfers. Moreover, we need to know which parameters promote or restrict dsRNA and siRNA uptake in a plant species-specific context at the “outside-inside” barrier and the “apoplast-symplast” interface, as well as in a pathosystem specific manner at the “plant-fungus” interface. However, the mechanism by which sprayed RNA overcomes the apoplast-symplast barrier is largely unknown. It is hypothesized that endocytosis and extracellular vesicles (EVs) play a crucial role in the uptake and translocation of HIGS- and SIGS-associated RNAs (Wytinck et al., 2020a; Koch and Wassenegger, 2021; Santos et al., 2021). Notably, recent data suggest that SIGS, in contrast to HIGS, might not involve EVs for siRNA delivery and uptake, at least in the *F. graminearum*-barley pathosystem (Schlemmer et al., 2021a,b). Based on insights into the mode of uptake, we have the chance to optimize and develop dsRNA and siRNA delivery and cellular uptake in the future.

## Significant Advances of RNA Spray Controlling: Insects

Host-induced gene silencing-mediated control of insect pests has been proven effective with an average of 50% conferred resistance (Koch and Wassenegger, 2021). Exogenous dsRNA and siRNA applications (e.g., feeding, injection, and oral delivery) to various insect pests and mites is routinely conducted to study gene function or identify RNAi targets (Lü et al., 2020; Mehlhorn et al., 2020, 2021; Máximo et al., 2020). In line with the above discussion about RNAs that remain on sprayed surfaces and those that enter plant cells, insect pests and mites can ingest dsRNA and siRNAs by chewing-biting or piercing-sucking feeding behaviors. In addition to the control of viruses and fungi, insect pests are also generally accessible to RNA sprays (Table 1). For example, a recent report demonstrated the efficiency of RNA sprays against *Henosepilachna vigintioctopunctata* (28-spotted ladybird) in the greenhouse (Wu et al., 2021). Spraying *E. coli*-expressed dsRNAs targeting the *ecdysone receptor* (*EcR*) gene onto the foliage of greenhouse-growing potato plants provoked the death of third and fourth instar larvae and reduced leaf damage (Wu et al., 2021). Confirming these results, another study showed exogenous dsRNA application as a promising alternative to chemical pesticides for controlling *H. vigintioctopunctata* (Lü et al., 2021). RNA sprays have also been shown to be effective in controlling the Colorado potato beetle, *Leptinotarsa decemlineata* (Mehlhorn et al., 2020). Even more importantly, the authors investigated geographical variation in RNAi sensitivity in the second instar larvae of 14 different European populations of field-collected *L. decemlineata* and found only minor variability in RNAi sensitivity between populations. This baseline study provides the first valuable insights on the broad applicability and transferability of RNA sprays over a geographic range in Europe (Mehlhorn et al., 2020). While the accessibility of phyllophagous pests by RNA sprays may seem trivial, a breakthrough was achieved by providing the first laboratory evidence that feeding dsRNA-coated oilseed rape buds to the pollen beetle *Brassicogethes aeneus* diminishes pollen beetle survival rate (Willow et al., 2021). This study is significant because many pathogens and pests that tremendously impact agriculture infect ears and buds rather than leaves.

Given the fact that we lack reliable results from field trials, or even from data generated under simulated field test conditions, lab-to-field transfer will require improvements of stability and adherence of dsRNA and siRNA to resist UV radiation and rain wash-off. For these purposes, encapsulation is pursued to guarantee and prolong the longevity of RNA sprays in the field. For example, *E. coli* is not only utilized for cost-effective dsRNA production but also represents a protective envelope for efficient dsRNA delivery (Lü et al., 2020; Máximo et al., 2020; Wang et al., 2021; Wu et al., 2021). Interestingly, encapsulation of dsRNA, if large enough, may prevent cellular uptake (plant intake) and thus shield dsRNA from plant DCL processing, as indicated by previous breakthroughs demonstrating that the expression of dsRNA in chloroplasts (DCL-free organelles) targeting the *ß-actin* gene of *L. decemlineata* (Colorado potato beetle) caused larval lethality (Zhang et al., 2015).

Targeting piercing-sucking insects by RNA spray requires cellular uptake and systemic distribution via the phloem. Thus, knowledge of the paths used by dsRNA and siRNA as SIGS inducers is a prerequisite for further developing and applying RNA sprays to the field. Given this assumption, previous reports revealed the systemic spread of sprayed RNAs (Koch et al., 2016; Konakalla et al., 2016; Kaldis et al., 2018; Biedenkopf et al., 2020). Spraying a fluorescent-labeled dsRNA onto barley leaves and subsequently examining longitudinal leaf sections revealed that the fluorescence was not confined to the apoplast but also was present in the symplast of phloem parenchyma cells, companion cells and mesophyll cells, as well as in trichomes and stomata (Koch et al., 2016). The finding that RNA biopesticides systemically spread through the phloem was confirmed by spraying fluorescent-labeled dsRNA followed by phloem sampling by stylectomy (excision of a stylet, typically that of an aphid) of the distal, non-sprayed leaf parts. Using CLSM, a green fluorescent signal was detected after cutting off the stylet tip of feeding aphids (Biedenkopf et al., 2020). Even more importantly, it was shown that sprayed dsRNA moved from barley leaves over stems to the root tissue within 3 days of spray treatment (Biedenkopf et al., 2020). This finding is especially interesting for the control of root pathogens because so far dsRNA applied to soil has immediately degraded (Dubelman et al., 2014; Parker et al., 2019; Bachman et al., 2020; Qiao et al., 2021). However, systemic distribution may result in dilution of SIGS signals; thus, further research is needed to prove the activity of dsRNA and siRNA in systemic tissues and to address the question of how much or how often RNA biopesticides need to be applied to confer robust and durable SIGS-based disease resistance. Given these challenges, previous studies have proposed utilizing the symbiosis of plants with bacteria, for example by engineering symbionts that serve as dsRNA vectors to maintain long-lasting dsRNA production *in planta* (Whitten et al., 2016; Whitten and Dyson, 2017). However, emerging data suggest that pest control via RNA sprays does not necessarily require plant passage for uptake and SIGS induction (Thairu et al., 2017; Niu et al., 2019; Linyu et al., 2021). Due to the limited uptake of dsRNA in hemiptera insects (Niu et al., 2019) originally developed and described gene silencing induction in aphids upon foliar spray on pea aphids themselves to verify potent RNAi targets in aphids. More recently, another confirmatory report used nanocarrier SPc (star polycation) transdermal delivery systems to verify gene silencing efficiency of selected RNAi targets in *Aphis gossypii* (Linyu et al., 2021). However, both studies imply sensitivity of RNA aphid sprays, which may circumvent plant passage and uptake from the phloem when applied in the field. Moreover, the development of formulations that allow transdermal delivery in a species-specific context is very valuable, especially in light of risk assessment and regulations (avoiding off-target effects).

We predict that nanomaterial-based formulations can solve major challenges regarding lab-to-field transitions by increasing stability and warranting specificity and selectivity. Not only surface stability, but also rather the ability to resist enzymatic degradation by RNase or extreme pH-values, which are prominent in saliva and gut of insects (Christiaens et al., 2014; Peng et al., 2020a,b). Given these challenges, a recent breakthrough was achieved, demonstrating that chitosan nanoparticles-mediated dsRNA delivery in *Helicoverpa armigera* protects from degradation by nucleases and insect gut pH (Kolge et al., 2021). Beyond shielding dsRNA from RNase degradation, the silencing of a dsRNA ribonuclease improved oral RNAi efficacy in the southern green stinkbug (Sharma et al., 2021). This finding indicates that the simultaneous application of protective agents such as RNase inhibitors or the silencing of dsRNases might encourage targeted detoxification mediated by P450s, ABC transporters and others (Wang et al., 2021). Notably, a nanomaterial-based formulation may help to increase selectivity not only by shielding dsRNA from the environment but also by providing target-site-specific RNA release (e.g., pH-dependent release kinetics), or selectivity facilitated by attractants that are incorporated in the envelope of (nano)-capsules following the proven attract and kill principle.

## Significant Advances of RNA Spray Silencing Plant Genes

Overcoming cuticle and cellular barriers is a prerequisite if RNA sprays are to substitute for conventional chemical herbicides or be used to study the gene function of plants. This is fundamentally different from RNA biopesticides that lay on plant, fruit or vegetable surfaces, in which cases sprays of naked dsRNA exhibit strong effects, for example, in controlling *B. cinerea* and *Botryotinia fuckeliana* (Wang et al., 2016; Islam et al., 2021; Qiao et al., 2021). However, previous attempts at spraying naked dsRNA to silence the expression of transgenes gave contrasting results, starting a controversial debate on the future herbicide uses of RNA sprays (Uslu and Wassenegger, 2020). For example, uptake of naked dsRNA and transgene silencing was demonstrated for the model plant *Arabidopsis thaliana* (Mitter et al., 2017; Dubrovina and Kiselev, 2019). However, attempts to silence transgene-expressed green fluorescence protein (GFP) in *Nicotiana benthamiana* by a high-pressure spray of naked dsRNA failed (Uslu et al., 2020). Notably, high-pressure sprays were specifically developed for RNAi applications and usage in *N. benthamiana*, resulting in transgene silencing (Dalakouras et al., 2016, 2018). Remarkably, based on their RNA-seq results the authors concluded that failure in transgene silencing correlates with the absence of dsRNA-derived specific siRNAs (Uslu et al., 2020). This finding raises the question of whether there was cellular uptake of high-pressure sprayed dsRNA, which is required for processing by plant DCLs. Notably, the spraying of 22 nt synthetic siRNA (matching the GFP sequence, position 164–187) as a positive control induced transgene silencing, as previously described (Dalakouras et al., 2016). Regarding this, another recent report showed induced methylation of the 35S promotor (which controls GFP transgene expression) upon 35S-dsRNA high pressure spray indicating cellular uptake of dsRNA (Dalakouras and Ganopoulos, 2021). Nevertheless, in contrast to siRNA spraying, cellular uptake of sprayed long dsRNAs seems to be less efficient, especially entering the nucleus to trigger RNA-directed DNA methylation is challenging. Moreover, another previous study demonstrated cellular uptake and plant DCL-mediated siRNA generation of a naked dsRNA sprayed onto barley leaves; however, this did not target a transgene (Koch et al., 2016). Nevertheless, these findings clearly illustrate how different plant species respond to RNA sprays, thus exemplifying the importance and urgency of research into the mechanistic basis (uptake, processing, and translocation) of RNA spray applications.

As much as spraying naked dsRNA highlights the simplicity of this technology, there are limits and restrictions that we have just begun to unravel. Given that sufficient RNA delivery is a continuing challenge in RNA spray applications, significant advances in nanomaterial-based formulations and the development of other smart delivery platforms have boosted technological development. Utilizing different biological, physical, and chemical-assisted delivery methods such as Bacterium-mediated RNAi (Goodfellow et al., 2019), high-pressure sprays (Dalakouras et al., 2016, 2018; Uslu et al., 2020), lipid nanoparticles, cationic polymers, cell-penetrating peptides and clay nanosheets has been shown to provide protection from nucleases and pH and to improve cellular uptake increasing plant resistance to pathogens and pests (Table 1). Recent studies have demonstrated efficient siRNA delivery into intact plants facilitated by nanomaterials such as carbon dots (Schwartz et al., 2020), single-walled carbon nanotubes (Demirer et al., 2019, 2020), DNA nanostructure carriers (Zhang et al., 2019, 2020) and gold-nanoclusters (Zhang et al., 2021; Table 1). Based on their small size (3.9 nm) carbon dots can pass through the cell wall (size exclusion limit 3–10 nm) (Carpita et al., 1979) mediating siRNA delivery into plant cells (Schwartz et al., 2020). Notably, passing through a plant cell wall is not the only prerequisite for efficient silencing. As discussed above, RNAi effectors face several barriers in reaching their mRNA cognates. Thus, cellular uptake requires overcoming the apoplast-symplast barrier mediated by endocytosis through the plasma membrane and subsequent release from endomembrane vesicles (Wytinck et al., 2020b). Given these later barriers, the properties of carbon dots are found to be suboptimal (Schwartz et al., 2020). Although carbon-dots-mediated siRNA delivery needs further improvement for efficient endogenous gene silencing, the delivery of dsRNAs over plant cell walls that reach the apoplast might be sufficient for controlling pathogens that require the uptake of unprocessed dsRNAs for SIGS induction (Koch et al., 2016; Gaffar et al., 2019). Due to their ultrasmall size (2 nm) and easier and faster synthesis, gold-nanoclusters (AuNCs) have been adapted for the delivery of siRNAs in mature plants, inducing efficient transgene as well as endogenous gene silencing in *N. benthamiana* (Zhang et al., 2021).

However, as naked dsRNA/siRNA delivery is restricted by largely unknown parameters that prevent RNA sprays from reaching their full potential, usage of different delivery methods, as advantageous and promising they may be, have limitations, as indicated above (for a detailed comparison of delivery platforms, see also: Zhang et al., 2021). Regarding this, identification of chemicals (e.g., Sortin1) that increased RNAi potency by enhancing siRNA accumulation and loading into AGOs may boost the development of powerful RNA biopesticide formulations (Jay et al., 2019). Moreover, while all of these studies provided proof-of-concept, the suitability of nanomaterial-based RNAi effector delivery as well as application of chemical enhancers of PTGS needs to be approved under field conditions. In addition, toxicity and biodegradability, together with applicability to other plant species and transferability for delivering dsRNA targeting foreign genes, needs further research in order to be verified.

## Conclusion and Future Perspectives

Despite the numerous proof-of-concept studies demonstrating the great potential underlying RNAi-based plant protection, especially GMO-free RNA sprays, we are still far from field applications or even product launches. Currently, we are facing complex and multi-layered challenges comprising technical-biological prospects (stability, selectivity, and broad applicability) as well as social-political demands (acceptance and regulations). To meet these challenges, significant advances in developing RNA sprays that guarantee stability in the field and minimize off-target risks for non-target organisms have been made, for example, by using nanomaterial-based formulations and the fusion of different technologies. However, we lack a sufficient data basis for understanding how nano-formulated RNA biopesticides will behave under field conditions. Moreover, we urgently require knowledge on durability, persistence and systemic effects of RNA sprays to develop recommendations for handling in the field. For example, when and how often do RNA biopesticides need to be applied or renewed to confer maximum protection? Based on the answer, we could calculate real costs for each pathosystem. To achieve broad applicability, which is often used as a selling point over GMO-based RNAi strategies (HIGS), we need to clarify the question of why RNA sprays work well for some pathosystems and not for others. If we know what determines SIGS efficiencies, we may have a starting point for developing solutions that demonstrate broad transferability. Elucidating the molecular mechanisms of uptake, processing and translocation of spray-delivered RNAs is crucial for making RNA sprays realistic and achievable in future field applications. In addition, emerging data that suggest extending RNA sprays to exploit the mechanisms of TGS that will allow epigenetic modifications of plant genes (Dalakouras and Vlachostergios, 2021) should be considered and discussed regarding the potential risk of introducing unintended off-target effects by modifying the plant’s epigenome (Dalakouras and Papadopoulou, 2020).

In contrast to lab experiments, where dsRNA and siRNA effects are tested in isolation and on small scales, field environments are unpredictable and highly dynamic. For example, the occurrence of pathogens and pests, as well as their coincidences, represents a major challenge for determining application time and frequencies. Moreover, plants grown under environmental conditions develop differently from plants grown in greenhouses, especially in terms of cuticle properties, which may impact adherence, stability, and cellular uptake. Thus, we need smart application and formulation strategies, such as multi-targeting and site-directed targeting, to ensure high efficacies on the species level. For example, combining RNA biopesticides with distinct molecular targets may not only confer stronger effects but also allow high efficiencies on a species-specific scale. In other words, the majority of highly effective dsRNAs and siRNAs target highly conserved genes, which bear high off-target risks. They are suitable candidates when conducting proof-of-concept studies but are inappropriate for field trials. Thus, identifying molecular targets on species level and their simultaneous combinatory (multi-targeting) applications may exhibit strong effects and at the same time minimize off-target risks. However, lab-to-field transitions require further precision and accuracy in off-target predictions and their controllability for adequate risk assessment. Currently, data requirements specific for RNA spray-based plant protection products are not yet in place under the regulatory frameworks for pesticides and plant protection products in the United States and EU (Dietz-Pfeilstetter et al., 2021). The OECD (Organization for Economic Cooperation and Development) organized a meeting on this question, which represented a major step forward in terms of testing regulation and research direction for future external dsRNA products (OECD, 2020). It is important to note that siRNAs display small gene regulatory units, which needs to be considered when developing directives that ensure appropriate risk assessment. Consistent with this, a recent report reviewed the evidence for and against the transfer of diet-derived miRNAs from plants, meat, milk and exosome and their putative molecular regulator roles as well as pharmacological opportunities for cross-kingdom regulation in the consuming organism (Mar-Aguilar et al., 2020; del Pozo-Acebo et al., 2021). The authors concluded that the transfer of miRNAs from the diet to the blood is still inconclusive and that the main source of controversy in plant studies is the lack of reproducibility of the findings. Currently, a risk assessment by the European Food Safety Authority (EFSA) classified RNA biopesticides as safe regarding low risks that sprayable RNAs pose for animals/humans (Olivier et al., 2018). The decisive argument was that oral uptake of RNAi products by consumers bears a low risk for interference with gene expression in humans, as too many biological and physical barriers have to be overcome (Schiemann et al., 2019; Kleter, 2020). However, so far, the available scientific data on environmental, consumer and user safety evaluations of RNA spray applications are still scarce. Preliminary data on the environmental fate (persistence and degradability) of sprayed dsRNA suggest a short shelf-life after application and absorption to soil (Parker et al., 2019; Bachman et al., 2020). However, ongoing nanomaterial-based attempts to increase stability may prolong environmental persistence and increase the risk to local ecosystems. In addition, it is still unclear whether and to what extent sprayed dsRNAs and siRNAs can accumulate along the food chain and how nanomaterials affect this accumulation. Thus, the environmental risk assessment of RNA biopesticides needs to be reinforced in order to facilitate their future placement on the market. Moreover, the length of the pesticide authorization process should be reduced to support a just transition. Given this, it is just a matter of time since first RNA biopesticides (e.g., Monsanto’s/Bayer’s BioDirect^TM^ technology) will access the market. Finally, we need to develop educational work and information campaigns for public outreach and transparency as early as possible.

## Author Contributions

Both authors contributed to wrote the manuscript and approved the submitted version.

## Conflict of Interest

The authors declare that the research was conducted in the absence of any commercial or financial relationships that could be construed as a potential conflict of interest.

## Publisher’s Note

All claims expressed in this article are solely those of the authors and do not necessarily represent those of their affiliated organizations, or those of the publisher, the editors and the reviewers. Any product that may be evaluated in this article, or claim that may be made by its manufacturer, is not guaranteed or endorsed by the publisher.

## Footnotes

1
https://www.rnaissanceag.net

2
http://www.globalengage.co.uk/pgc/docs/PosterMaxwell.pdf
